# Combined 3D-QSAR Modeling and Molecular Docking Studies on Pyrrole-Indolin-2-ones as Aurora A Kinase Inhibitors

**DOI:** 10.3390/ijms12031605

**Published:** 2011-03-01

**Authors:** Yong Ai, Shao-Teng Wang, Ping-Hua Sun, Fa-Jun Song

**Affiliations:** 1 College of Pharmacy, South Central University for Nationalities, 708 Minyuan Road, Wuhan 430074, China; E-Mails: aiyong0508@126.com (Y.A.); wst418638862@sohu.com (S.-T.W.); 2 Guangdong Province Key Laboratory of Pharmacodynamic Constituents of TCM and New Drugs Research, College of Pharmacy, Jinan University, Guangzhou 510632, China; E-Mail: Pinghuasunny@163.com; 3 College of Life Sciences, South Central University for Nationalities, 708 Minyuan Road, Wuhan 430074, China

**Keywords:** 3D-QSAR, CoMFA, CoMSIA, Docking, pyrrole-indoline-2-ones, Aurora kinases

## Abstract

Aurora kinases have emerged as attractive targets for the design of anticancer drugs. 3D-QSAR (comparative molecular field analysis (CoMFA) and comparative molecular similarity indices analysis (CoMSIA)) and Surflex-docking studies were performed on a series of pyrrole-indoline-2-ones as Aurora A inhibitors. The CoMFA and CoMSIA models using 25 inhibitors in the training set gave *r^2^_cv_* values of 0.726 and 0.566, and *r^2^* values of 0.972 and 0.984, respectively. The adapted alignment method with the suitable parameters resulted in reliable models. The contour maps produced by the CoMFA and CoMSIA models were employed to rationalize the key structural requirements responsible for the activity. Surflex-docking studies revealed that the sulfo group, secondary amine group on indolin-2-one, and carbonyl of 6,7-dihydro-1*H*-indol-4(5*H*)-one groups were significant for binding to the receptor, and some essential features were also identified. Based on the 3D-QSAR and docking results, a set of new molecules with high predicted activities were designed.

## 1. Introduction

The Aurora kinases are a family of highly conserved serine/threonine protein kinases that play a key role in regulating many pivotal processes of mitosis and completion of cell division [1–5]. The two major Aurora kinases, Aurora A and Aurora B, play distinct roles in mitosis, though they are very closely related in kinase domain sequence (71% identical) and have the identical residues lining the binding pocket for the ATP adenine ring [6]. Aurora A is involved in centrosome maturation and separation, bipolar spindle assembly, and mitotic entry, while Aurora B is essential for accurate chromosome segregation and cytokinesis [7]. In recent years, Aurora A and B have been actively pursued as anticancer targets for the discovery of new cancer chemotherapeutics [8]. Aurora A has especially been identified as a colon-cancer-associated kinase that is overexpressed in a wide range of human tumors such as breast, colorectal, ovarian, as well as glioma [9,10]. Thus, targeted inhibition of Aurora A has become an attractive therapeutic strategy in cancer therapy, and more than 10 Aurora inhibitors have entered early clinical assessment [11,12].

A series of pyrrole-indoline-2-ones with Aurora A inhibitory activities were reported [13]. These pyrrole-indoline-2-ones, with excellent Aurora A inhibitory activities, were designed and synthesized by sharing a similar scaffold with Hesperadin [14]. In the present study, three-dimensional quantitative structure-activity relationship (3D-QSAR) methods along with docking approaches were used to explore the structure-activity relationship (SAR) of these pyrrole-indoline-2-ones. 3D-QSAR methods, comparative molecular field analysis (CoMFA) and comparative molecular similarity indices analysis (CoMSIA), were performed to foresee the activities of these pyrrole-indoline-2-ones and offered the regions where interactive fields (steric, electrostatic, hydrophobic, hydrogen bond donor and hydrogen bond acceptor fields) may increase or decrease the activity. Surflex-Docking was applied to study the interactions between 35 pyrrole-indoline-2-ones and Aurora A. These developed models can help understanding the SAR of the pyrrole-indoline-2-ones and can also serve as a valuable guide for the design of novel inhibitors with robust potency.

Furthermore, we have designed a number of new pyrrole-indoline-2-ones derivatives by utilizing the structure information obtained from the CoMFA and CoMSIA models, which exhibit excellent predictive potencies. Moreover, based on the admirable performance of docking studies, the predicted activities of these newly designed molecules may be reliable.

## 2. Materials and Methods

### 2.1. Data Sets

All of the 35 compounds and associated data involved in this study were obtained from literature [13]. The inhibitory activity data were reported as IC_50_ against Aurora A. The IC_50_ values were converted into pIC_50_ according to the formula in Equation 1 [15]. In CoMFA and CoMSIA models, the dataset was randomly divided into training and test sets including 25 and 10 molecules, respectively. The structures of the molecules are shown in Table 1 and associated inhibitory activities are shown in Table 2, where pIC_50_ values for 35 inhibitors ranged from 5.611 to 8.073.

(1) $${\text{pIC}}_{50}=-\text{Log}\;{\text{IC}}_{50}$$

### 2.2. Molecular Modeling and Database Alignment

Molecular modeling and database alignment were performed by using the molecular modeling package SYBYL 8.1 Tripos, Inc. [16]. The three-dimensional structures of all pyrrole-indoline-2-ones were constructed by using the Sketch Molecule module. Energy minimization of each structure was performed using the SYBYL energy minimizer Tripos force field and Gasteiger-Hückel charge. The maximum iterations for the minimization was set to 2000. The minimization was terminated when the energy gradient convergence criterion of 0.05 kcal/mol·Å was reached [17].

**Figure 1.**
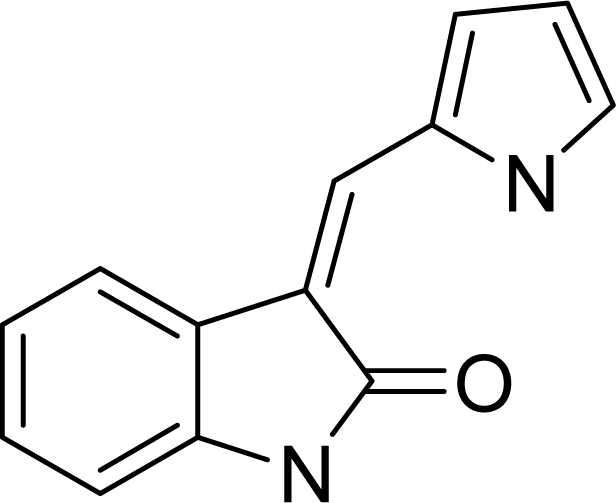
Common substructure used for alignment.

**Figure 2.**
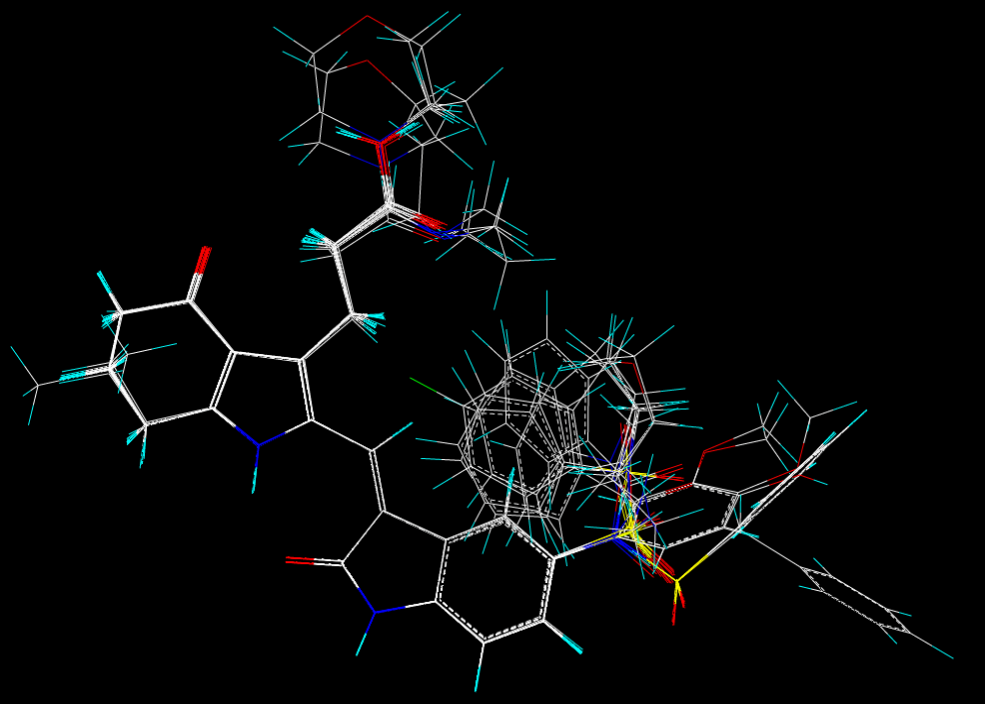
Alignment of the compounds used in the training set.

Molecular alignment was considered as one of the most sensitive parameters in 3D-QSAR analyses [18]. The quality and the predictive ability of the model are directly dependent on the alignment rule [19]. In this paper, all of the structures were aligned into a lattice box by fitting with (*Z*)-3-((1*H*-pyrrol-2-yl)methylene)indolin-2-one (Figure 1) as a common structure using compound **20** as a template, which was the most active compound. The aligned molecules are shown in Figure 2.

### 2.3. CoMFA and CoMSIA Setup

CoMFA is a widely used 3D-QSAR method, which relates the biological activity of a series of molecules with their steric and electrostatic fields. The CoMFA descriptor fields were calculated at each lattice with a grid spacing of 1 Å and extending to 4 Å units in all three dimensions within the defined region [20]. The Van Der Waals potentials and Coulombic terms, which represent steric and electrostatic fields, respectively, were calculated by using the standard Tripos force field. In the CoMFA method, a sp^3^ hybridized carbon atom with a charge of 1e was used as a probe atom, the energy values of the steric and electrostatic fields were truncated at 30 kcal/mol [21].

The steric, electrostatic, hydrophobic, hydrogen bond donor and hydrogen bond acceptor CoMSIA potential fields were calculated at each lattice intersection of a regularly spaced grid of 1 Å and extending to 4 Å using a probe atom with radius 1.0 Å, +1.0 charge, and hydrophobic and hydrogen bond properties of +1. The attenuation factor was set to the default value of 0.3 [22].

### 2.4. Regression Analysis and Models Validation

The partial least-squares (PLS) approach, an extension of multiple regression analysis, was applied to linearly correlate the CoMFA and CoMSIA fields to the pIC_50_ values. CoMFA and CoMSIA descriptors were used as the independent variables. Column filtering was used at the default value of 2.0 kcal/mol in the cross-validation part.

The cross-validation analysis was performed using the leave-one-out (LOO) method in which one molecule was omitted from the dataset. The activity of the omitted molecule was then predicted by using the model derived from the rest of the dataset [23]. The leave-one-out (LOO) cross-validation method could check the predictivity of the obtained model and identify the optimum number of components (ONC). Thus, the optimum number of components (ONC) was the number of components that led to the highest cross-validated correlated correlation coefficient *r*^2^ (*r*^2^_cv_) [24]. Finally, the CoMFA and CoMSIA models were generated using non-cross-validated PLS analysis with the optimum number of components (ONC) determined by the cross-validation.

### 2.5. Predictive Correlation Co-efficient (r^2^_pred_)

The predictive abilities of 3D-QSAR models were validated by predicting the activities of a test set of 10 compounds that were not included in the training set. These molecules were aligned to the template and their pIC_50_ values were predicted by the produced models which were obtained using the training set. The predictive correlation coefficient (*r*^2^_pred_), based on the molecules of the test set, was calculated using Equation (2):

(2) $$r_{\text{pred}}^{2}=(\text{SD-PRESS})/\text{SD}$$

In this equation, SD is the sum of the squared deviations between the inhibitory activities of the test set and the mean activity of the training molecules and PRESS is the sum of squared deviations between predicted and actual activity values for each molecule in the test set [25].

### 2.6. Molecular Docking

The Surflex-Dock was applied to study molecular docking by using an empirical scoring function and a patented search engine to dock ligands into a protein’s binding site [16]. The crystal structure of Aurora A was retrieved from the RCSB Protein Data Bank (PDB entry code: 2X6E). The ligands were docked into corresponding protein’s binding site by an empirical scoring function and a patented search engine in Surflex-Dock [16]. All ligands and water molecules in Aurora A 2X6E have been deleted and the polar hydrogen atoms were added to 2X6E. Protomol, a representation of a ligand making every potential interaction with the binding site, was applied to guide molecular docking. Protomols could be established by three manners: (1) Automatic: Surflex-Dock finds the largest cavity in the receptor protein; (2) Ligand: a ligand in the same coordinate space as the receptor; (3) Residues: specified residues in the receptor [26,27].

In this paper, the automatic docking was applied. The Aurora A structure was utilized in subsequent docking experiments without energy minimization. Other parameters were established by default in the software. Surflex-Dock scores (total scores) were expressed in –log_10_(K_d_) units to represent binding affinities. Then, the MOLCAD (Molecular Computer Aided Design) program was employed to visualize the binding mode between the protein and ligand. MOLCAD calculates and exhibits the surfaces of channels and cavities, as well as the separating surface between protein subunits [20–22]. MOLCAD program provides several types to create a molecular surface [16]. The fast Connolly method using a marching cube algorithm to engender the surface was applied in this work, thus the MOLECAD Robbin and Multi-Channel surfaces program exhibited with copious potentials were established. Moreover, Surflex-Dock total scores, which were expressed in –log_10_(*K*_d_) units to represent binding affinities, were applied to estimate the ligand-receptor interactions of newly designed molecules.

## 3. Results and Discussions

### 3.1. CoMFA and CoMSIA Models

**Figure 3.**
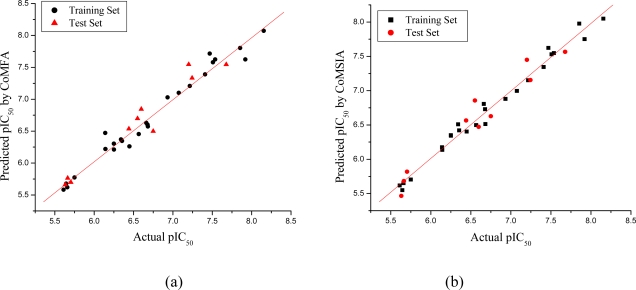
Graph of actual *versus* predicted pIC_50_ of the training set and the test set using CoMFA (**a**) and CoMSIA (**b**).

The statistical parameters for the CoMFA and CoMSIA models are given in Table 3. For the CoMFA model, partial least squares (PLS) regression produced a excellent cross-validated correlation coefficient (*r^2^_cv_*) of 0.726 (>0.5) with an optimized component of 6, which suggesting that the model is reliable and it should be a useful tool for predicting the IC_50_ values. The non cross-validated PLS analysis gave a high correlation coefficient (*r^2^*) of 0.972, *F* value of 105.300 and a low standard error estimate (SEE) of 0.144. The contributions of steric and electrostatic fields to this model were 0.457 and 0.543, respectively. The predictive correlation coefficient (*r*^2^_pred_) value based on molecules of the test set was 0.937 for the CoMFA model. The actual and predicted pIC_50_ values of the training set and test set by the model are given in Table 2. The relationship between actual and predicted pIC_50_ of the training set and test set compounds of the CoMFA model is illustrated in Figure 3a, where almost all points are located on the diagonal line.

For the CoMSIA model, the statistical parameters revealed that steric, electrostatic, hydrophobic, hydrogen bond donor and acceptor features significantly influence the activity of the inhibitors. The CoMSIA model gave a cross-validated correlation coefficient (*r^2^_cv_*) of 0.566 (>0.5) with an optimized component of 6, which suggested that the model is reliable and should be a useful tool for predicting the IC_50_ values. The non cross-validated PLS analysis gave a high correlation coefficient (*r^2^*) of 0.984, F value of 181.398 and a low error estimate (SEE) of 0.110. The contributions of steric, electrostatic, hydrophobic, hydrogen bond donor and acceptor fields were 0.152, 0.169, 0.243, 0.183 and 0.252, respectively. The predictive correlation coefficient (*r*^2^_pred_) value based on molecules of the test set was 0.948 for the CoMSIA model. The actual and predicted pIC_50_ values and residual values for training set and test set compounds are given in Table 2. The graph of actual activity *versus* predicted pIC_50_ of the training set and test set is illustrated in Figure 3b, where almost all points are located on the diagonal line.

### 3.2. CoMFA and CoMSIA Contour Maps

**Figure 4.**
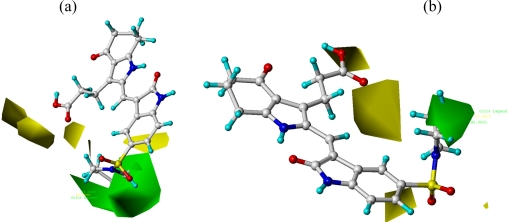
Contour maps of CoMFA (**a**) and CoMSIA (**b**) analysis in combination with compound **20.** Steric fields: green contours (80% contribution) indicate regions where bulky groups increase activity, while yellow contours (20% contribution) indicate regions where bulky groups decrease activity. Compound **20** is depicted in ball and stick representation, colored by atom type (white C, blue N, red O, cyan H).

**Figure 5.**
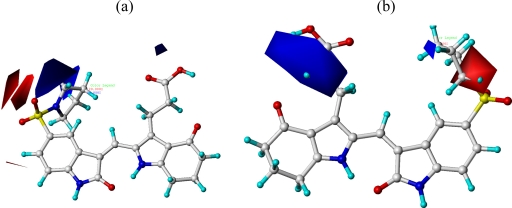
Contour maps of CoMFA (**a**) and CoMSIA (**b**) analysis in combination with compound **20.** Electrostatic fields: blue contours (80% contribution) represent regions where electron-donating groups increase activity, while red contours (20% contribution) represent regions where electron-withdrawing groups increase activity. Compound **20** is depicted in ball and stick representation, colored by atom type (white C, blue N, red O, cyan H).

**Figure 6.**
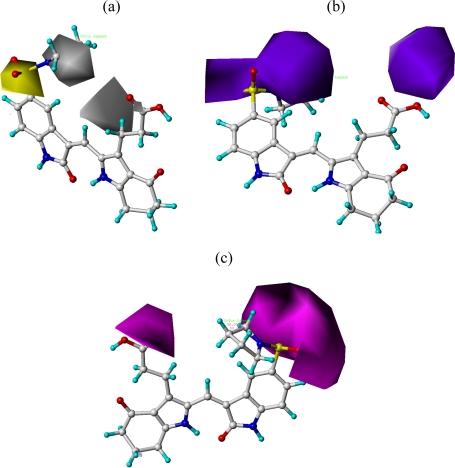
Contour maps of CoMSIA analysis in combination with compound **20**. Hydrophobic fields (**a**), the yellow and white contours (80% and 20% contributions) indicate favorable and unfavorable hydrophobic groups; Hydrogen bond donor contour map (**b**), the cyan and purple contours (80% and 20% contributions) indicate favorable and unfavorable hydrogen bond donor groups; Hydrogen bond acceptor contour map (**c**), the magenta and red contours (50% and 50% contributions) indicate favorable and unfavorable hydrogen bond acceptor groups. Compound **20** is depicted in ball and stick representation, colored by atom type (white C, blue N, red O, cyan H).

The results of the CoMFA and CoMSIA models were visualized through contour maps. These maps showed regions in 3D space where variation in specific molecular properties increased or decreased the activity. The molecular fields around the most active compound **20** are displayed in Figures 4–6, accordingly. These contour maps are significant for drug design, as they showed regions in 3D space where modifications of the molecular fields strongly correlated with concomitant changes in biological activity.

The steric contour map of CoMFA is shown in Figure 4a, which was almost the same as the corresponding CoMSIA steric contour map (Figure 4b). Compound **20** was selected as a reference molecule. The steric field was represented by green and yellow contours, in which green contours indicate regions where presence of bulky steric groups was favored and should enhance inhibitory activity of molecules, while the yellow contours represent regions where occupancy of steric groups was unfavorable. As shown in Figure 4, the presence of the green contour around the R^1^ position suggested that a bulky group at this region would be favorable. By checking up all the R^1^ modified compounds, it was found that derivatives **07**–**08** have the activity order of **07** (R^1^ = Br) > **08** (R^1^ = NO_2_); compounds **13**, **14**, **17** have the activity order of **14** (R^1^ = −SO_2_CH_2_CHCH_2_) > **13** (R^1^ = −SO_2_C_2_H_5_) > **17** (R^1^ = −SO_2_NH_2_); compounds **17**–**19** have the activity order of **20** (R^1^ = sulfo-pyrrolidine) > **19** (R^1^ = −SO_2_N(CH_3_)_2_) > **18** (R^1^ = −SO_2_NHCH_3_) > **17** (R^1^ = −SO_2_NH_2_); compounds **23**–**26** have the activity order of **23** (R^1^ = −NHSO_2_C_2_H_5_) < **24** (R^1^ = −NHSO_2_-benzene), **25** (R^1^ = −NHSO_2_-CH_2_-benzene) < **26** (R^1^ = −NHSO_2_-benzene). These were satisfactory according to the steric contour map. The R^2^ was surrounded by three yellow contours, which suggested a bulky group at this region would decrease the inhibitory activity. This may explain why compounds **1**–**2**, **5**, which possessed a relative bulky group (e.g., −COOEt) at R^1^, showed significantly decreased activities than other compounds with a relatively minor substituent at R^2^. For instance, derivative **24** bearing a carboxy group at R^2^ exhibited improved potency than compound **26** with an ethoxycarbonyl at this position. Furthermore, compound **20** with carboxyl group at the R^2^ position was the most inactive compound.

The electrostatic field contour maps of CoMFA and CoMSIA are shown in Figure 5a and b, respectively. Compound **20** was selected as a reference molecule again. The electrostatic field is indicated by blue and red contours, which demonstrate the regions where electron-donating group and electron-withdrawing group would be favorable, respectively. In the electrostatic field, two blue contours around the terminal of R^1^ and two red contours at the middle of the R^1^ revealed that the electron-donating substituents at the terminal of the R^1^ and the electron-withdrawing groups at the middle of the R^1^ were essential for the inhibitory activity. Take the compounds **13**–**22** and **24**–**26** (R^1^ = 4-CF_3_-benzyl) for an example, the strong electron-withdrawing sulfo, and sulfanilamido group at the middle of R^1^ and electron-donating Et, −NH_2_, −NHCH_3_, −N(CH_3_)_2_, 1-pyrrolidine, 1-piperidine, and 4-morpholine groups at the terminal of R^1^ in these compounds resulted in significantly increased activity. The blue contour near the chain between 6,7-dihydro-1*H*-inden-4(5*H*)-one and R^2^ suggested the electron-donating group (-CH_2_CH_2_-) at this position may be essential for potency. All of the derivatives involved in this study possessed a -CH_2_CH_2_- group at this site, which revealed the extreme importance of the electron-donating substituent.

In hydrophobic fields, yellow and white contours highlighted areas where hydrophobic and hydrophilic properties were favored. In Figure 6a, the yellow contour around the chain of R^1^ position indicated that a hydrophobic substituent would benefit the potency. Most of the derivatives involved in this study possessed a hydrophobic group at this site, which revealed the extreme importance of the hydrophobic substituent. Furthermore, the actual IC_50_ of these compounds was basically 10-fold than those without a hydrophobic group. A huge white contour near the terminal of R^1^ site suggested that a hydrophilic group may be favored. This may explain why derivative **20** with a relatively more hydrophilic N atom at this position exhibited better potencies than compounds **25**–**33**. Another white contour around R^2^ position demonstrated that a hydrophilic substituent carboxyl would be favorable. Most of the compounds possessed a hydrophilic substituent at this site, except compounds **1**–**2** with a hydrophobic −OOC_2_H_5_ at R^2^, which displayed lower activity than compounds **3**–**25**.

In hydrogen bond donor field, the cyan and purple contours indicated favorable and unfavorable hydrogen bond donor groups. In Figure 6b, the two bulk purple contours near the R^1^ position revealed that hydrogen bond acceptor groups may benefit the potency. In fact the sulfo group and quaternary amine atom at this position acted as hydrogen bond acceptor by forming H-bonds with residues of the ATP binding site of Aurora A. This may explain why compounds **19**–**22** displayed relatively better activities. Likewise, a bulk purple contour near R^2^ revealed that they acted as hydrogen bond acceptor by forming H-bonds with residues of the ATP binding site of Aurora A. Most of the derivatives involved in this study possessed a hydrogen bond acceptor group (carboxyl) at this site, which indicated the extreme importance of the hydrogen bond acceptor group substituent.

In hydrogen bond acceptor field, the magenta and red contours identified favorable and unfavorable positions for hydrogen bond acceptors. In Figure 6c, two bulk red contours around the R^1^ and R^2^ indicated that a hydrogen bond acceptor substituent at these sites would increase the activity. The inference obtained by Figure 6c satisfactorily matched the hydrogen bond donor contour map.

### 3.3. Docking Analysis

Surflex-Dock was applied to investigate the binding mode between these pyrrole-indoline-2-ones and Aurora A. In this paper, Surflex-Dock could also serve to inspect the stability of 3D-QSAR models previous established. To visualize secondary structure elements, the MOLCAD Robbin surfaces program was applied. Furthermore, the MOLCAD surface of ATP site was also developed and displayed with cavity depth (CD), electrostatic potential (EP) to further explore the interaction between these inhibitors and the receptor. The most potent inhibitor **20** was selected for more detailed research.

**Figure 7.**
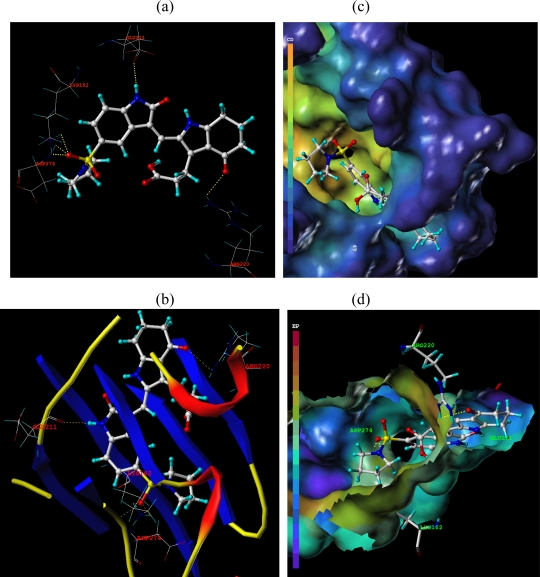
Docked binding modes of compound **20** in the ATP binding site of Aurora A (PDB code 2X6E). In each panel, compound **20** is shown as ball and stick representation; hydrogen bonds are shown as dashed yellow line; residues are shown as line representation. (**a**) Key residues (additional residues) and hydrogen bonds are distinctly labeled; (**b**) the MOLCAD ribbon surfaces: Alpha helices are shown as helices or cylinders; beta sheets are shown as arrows; the loop regions as tubes; key residues and hydrogen bonds are labeled; (**c**) The MOLCAD cavity depth potential surface: cavity depth color ramp ranges from blue (low depth values = outside of the pocket) to light red (high depth values = cavities deep inside the pocket); (**d**) The MOLCAD electrostatic potential surface: the color ramp for EP ranges from red (most positive) to purple (most negative).

In Figure 7a, the hydrogen bonding (dashed lines) interactions between the reference compound **20** with highest inhibitory activity is shown and the key residues (Lys162, Asp274, Glu211, and Arg220) of the ATP site of Aurora A (PDB code 2X6E) are labeled. A total of five hydrogen bonds were formed: the sulfo at R^1^ position acted as the hydrogen bond acceptor and formed two H-bonds with the secondary amino group of the Tys162 residue, and a H-bond with the secondary amino group of Asp274; the carbonyl substituent on the 6,7-dihydro-1*H*-inden-4(5*H*)-one scaffold in compound **20** also acted as the hydrogen bond acceptor and formed H-bond with primary amino group of Arg220 residue; the secondary amino on the 6,7-dihydro-1*H*-inden-4(5*H*)-one in compound **20** acted as H-bond donor and formed H-bond with carbonyl group of Glu211. These results observed by Figure 7a satisfactorily matched the observation taken from the CoMSIA hydrogen bond donor contour map.

In Figure 7b, the secondary structure of the ATP pocket within compound **20** is depicted: alpha helices are displayed as helices or cylinders, while beta sheets are shown as arrows and the loop regions as tubes. The key residues and hydrogen bonds (dashed lines) are labeled.

In Figure 7c, the MOLCAD Multi-Channel cavity depth potential surfaces structure of the binding site within the compound **20** is displayed and the cavity depth color ramp ranged from blue (low depth values = outside of the pocket) to light red (high depth values = cavities deep inside the pocket). In Figure 7c, the R^1^ position of compound 20 is observed in a blue area, revealing that this position was embedded deep inside the ATP pocket. It can be simply inferred that a bulky group at R^1^ position may be favorable. Since the R^2^ site was oriented to a light red area, which illustrated a minor group was anchored into a favorable region, this suggests that minor groups may benefit the potency. The observation obtained by Figure 7c satisfactorily matched the corresponding CoMFA and CoMSIA steric contour maps.

In Figure 7d, the MOLCAD electrostatic potential surface of the binding region was demonstrated with the color ramp for EP ranging from red (most positive) to purple (most negative). The 1-pyrrolidinyl group at the terminal of R^1^ was found in a blue area, which indicated that electron-donating properties at this site were essential for the potency; the sulfo group was in a yellow area, which suggested that electron-withdrawing properties would be favored; the -CH_2_CH_2_- chain between 6,7-dihydro-1*H*-inden-4(5*H*)-one and carboxyl was anchored in a blue area which suggested that an electron-donating substituent at this position would be essential for the potency. These results were well compared with the corresponding CoMFA and CoMSIA electrostatic contour maps.

### 3.4. Design for New Molecules

**Figure 8.**
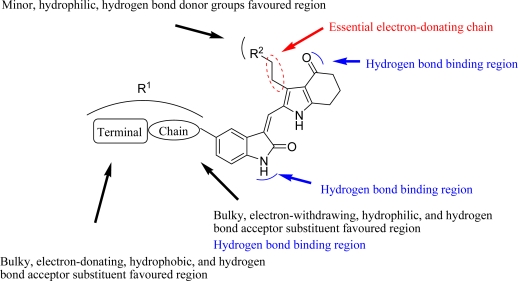
Structure-activity relationship revealed by present studies.

The detailed contour map analysis of both COMFA and CoMSIA models and the docking analysis empowered us to identify structural requirements for the observed inhibitory activity (Figure 8). In detail, bulky, electron-donating, hydrophobic, and hydrogen bond acceptor substituents at the terminal of R^1^ (e.g., pyrrolidine) would increase activity; bulky, electron-withdrawing, hydrophilic, and hydrogen bond acceptor substituents at the chain of the R^1^ (e.g., sulfo group) are favored for inhibitory activity; minor, hydrophilic, hydrogen bond donor groups at the R^2^ (e.g., carboxyl) may benefit potency. Moreover, the electron-donating chain (-CH_2_CH_2_-) between pyrrole and R^2^ may be essential for the activity of the inhibitors. The chain of the R^1^, indolin-2-one, and 6,7-dihydro-1H-indol-4(5*H*)-one groups were crucial for binding to ATP pocket of Aurora A.

**Figure 9.**
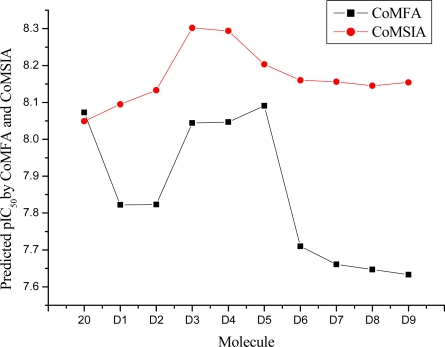
Predicted pIC_50_ values of newly designed molecules using CoMFA and CoMSIA.

**Figure 10.**
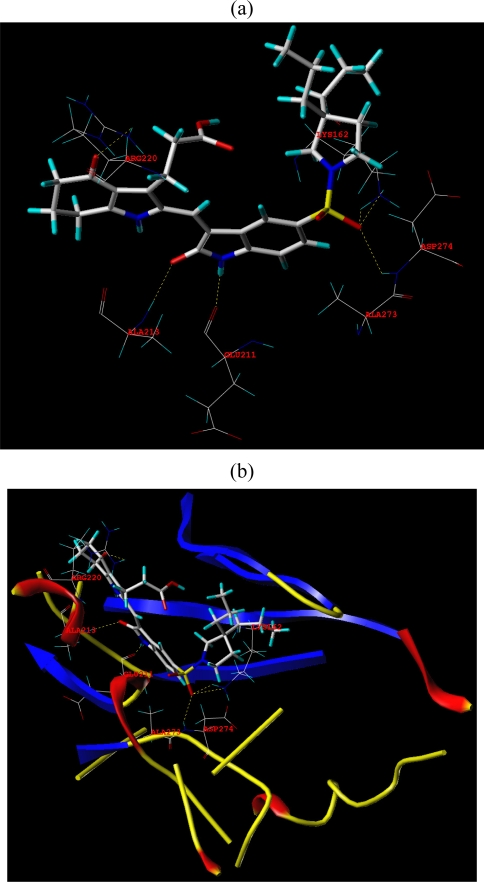
Docked binding modes of compound **D8** in the ATP binding site of Aurora A. Compound **D8** is shown as stick representation; hydrogen bonds are shown as dashed yellow lines; residues are shown as line representation. (**a**) Key residues (additional residues) and hydrogen bonds are distinctly labeled. (**b**) Alpha helices are shown as helices or cylinders; beta sheets are shown as arrows; the loop regions as tubes; key residues and hydrogen bonds are labeled.

Based on QSAR and docking results, inhibitor **20,** with the highest activity, was taken as a template to design new compounds. A set of nine new compounds with high predicted activity were designed and assessed (Table 4), and the graphs of their predicted pIC_50_ values *versus* the most active compound **20** are shown in Figure 9. After energy minimization, the nine new compounds were docked into the ATP binding site of Aurora A. The total scores of these compounds were higher than that of the template molecule (Table 4). The designed molecule **D8** was selected for more detailed investigation as one more H-bond from carbonyl on indolin-2-one group with Ala213 can be observed in Figure 10, where the compound **D8** with the highest surflex-dock total score were docked into the ATP pocket of Aurora A.

## 4. Conclusion

We employed 3D-QSAR and docking methods to explore the structure-activity relationship of a series of pyrrole-indoline-2-ones as Aurora inhibitors. The 3D-QSAR models described herein possessed excellent consistency. The predictive ability of the models were manifested in the good correlation between actual and predicted pIC_50_ values for the test molecules. The CoMFA and CoMSIA contour maps as well as the docking results provided enough information to understand the structure-activity relationship, identify structural features influencing the inhibitory activity, and furthermore, design new molecules. A number of novel pyrrole-indoline-2-ones derivatives were designed by using the SAR taken from the present study. The predicted activities of these newly designed pyrrole-indoline-2-ones may be reliable. The correlation of the results obtained from 3D-QSAR and docking studies can serve as a useful guideline for the further modification of pyrrole-indoline-2-ones that function as Aurora A inhibitors.

## Figures and Tables

**Table 1.** The structures of the training and test set molecules.

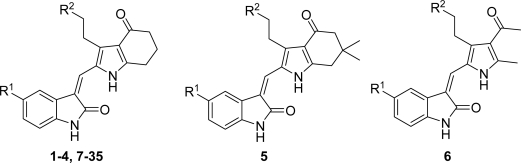		
**Compound No.**	**Substituent**	
	**R^1^**	**R^2^**
**1**	H	COOEt
**2**	F	COOEt
**3**	H	COOH
**4**	F	COOH
**5**	F	COOH
**6**	F	COOH
**7**	Br	COOH
**8**	NO_2_	COOH
**9**	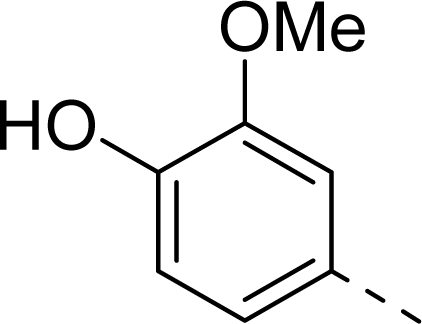	COOH
**10**	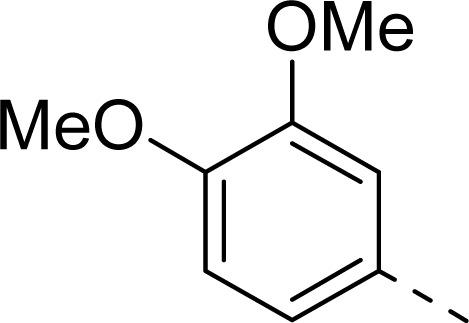	COOH
**11**	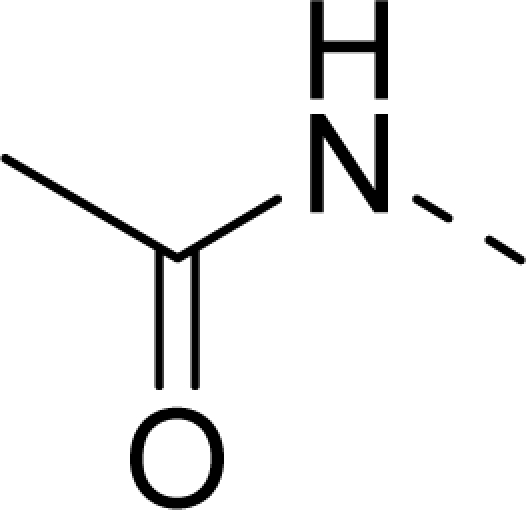	COOH
**12**	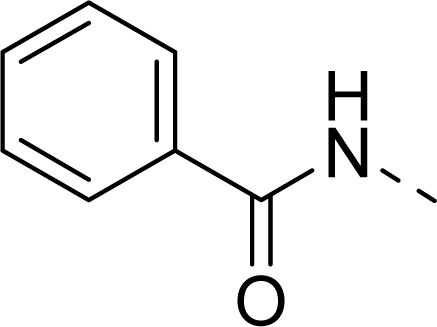	COOH
**13**	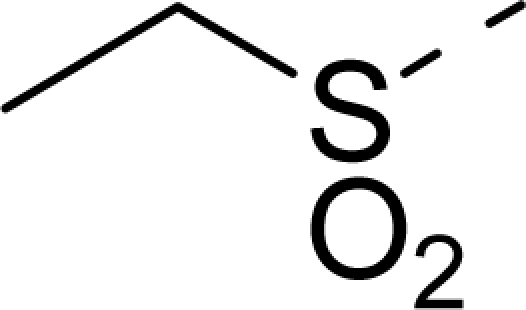	COOH
**14**	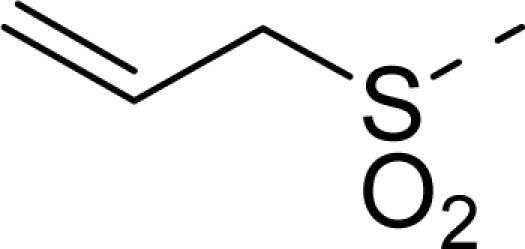	COOH
**15**	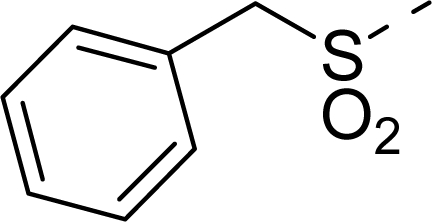	COOH
**16**	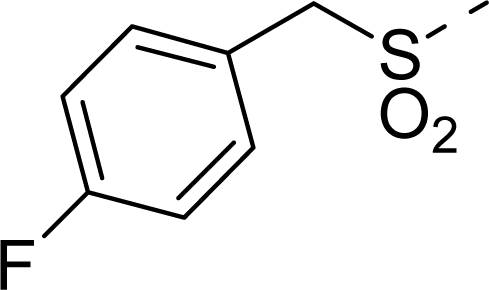	COOH
**17**	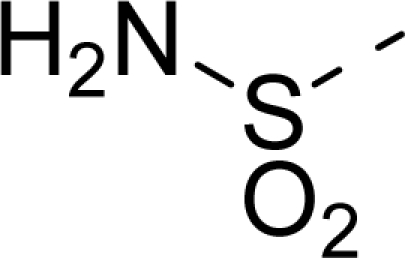	COOH
**18**	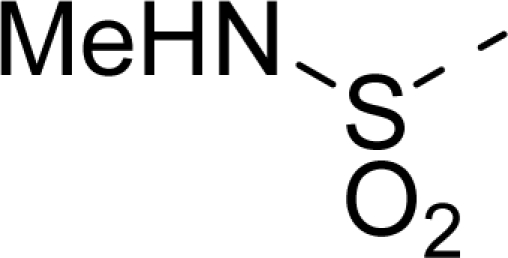	COOH
**19**	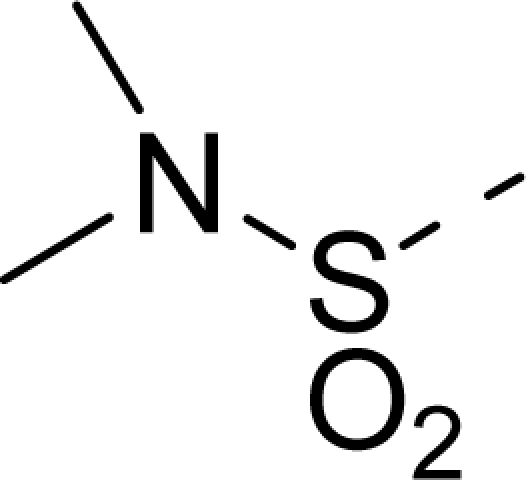	COOH
**20**	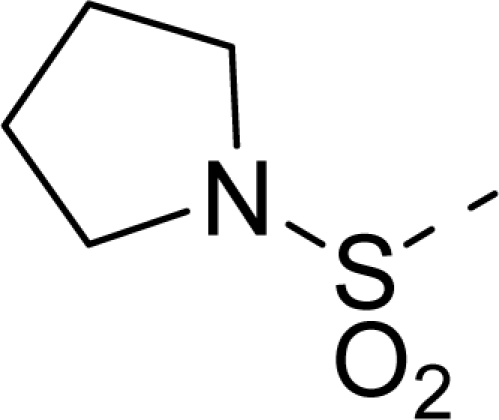	COOH
**21**	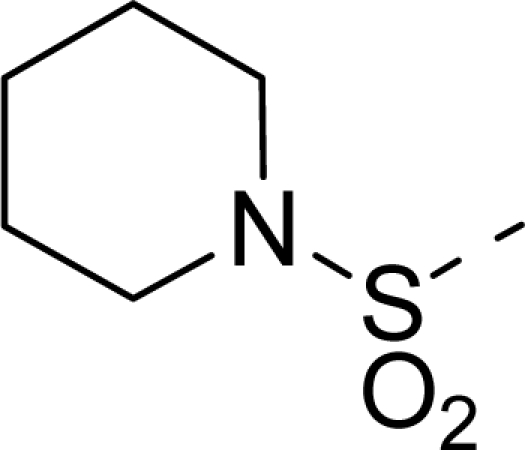	COOH
**22**	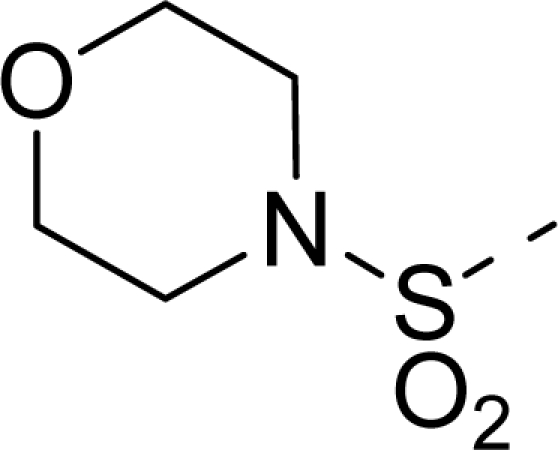	COOH
**23**	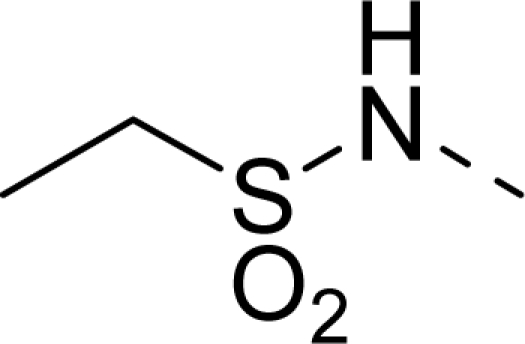	COOH
**24**	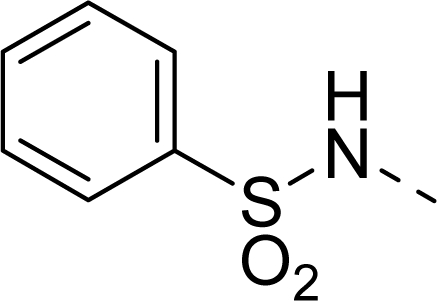	COOH
**25**	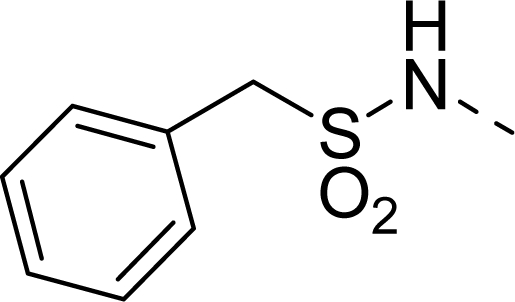	COOH
**26**	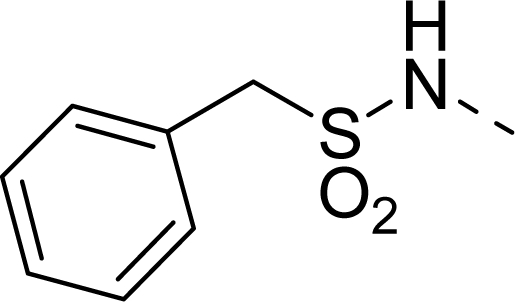	COOEt
**27**	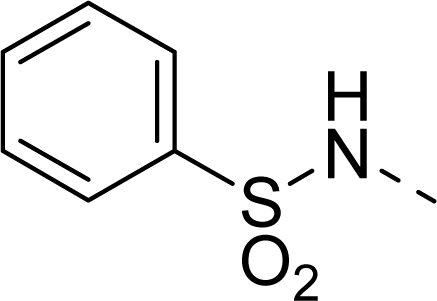	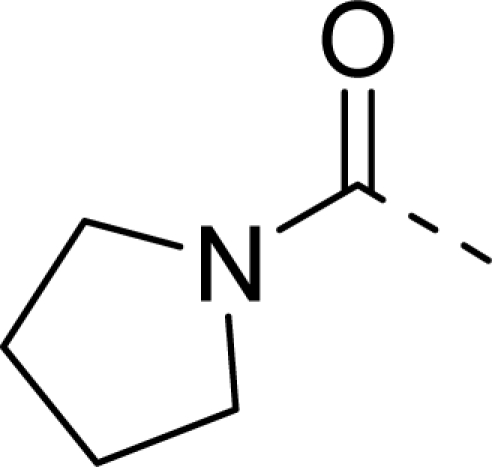
**28**	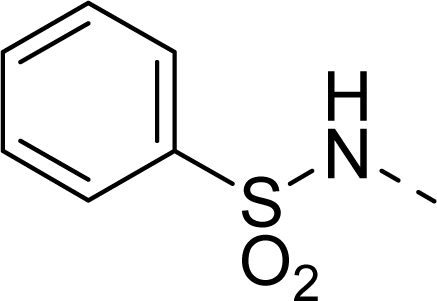	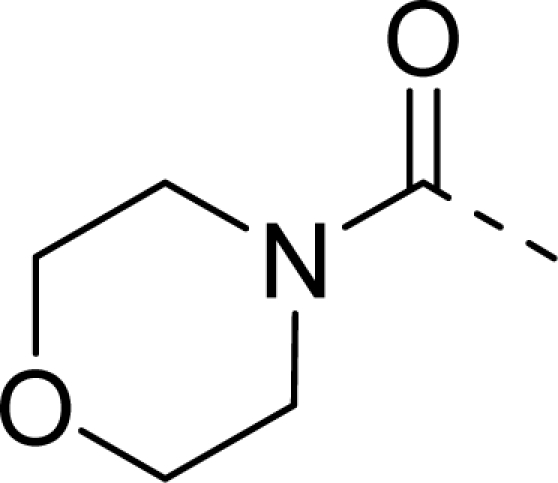
**29**	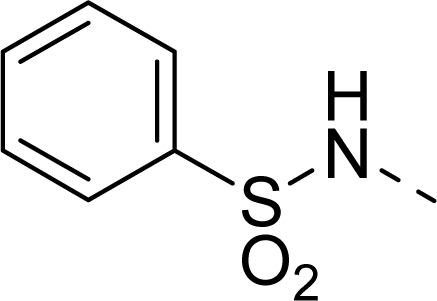	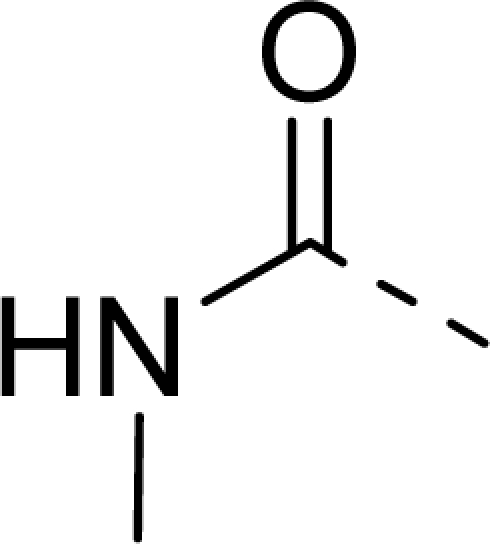
**30**	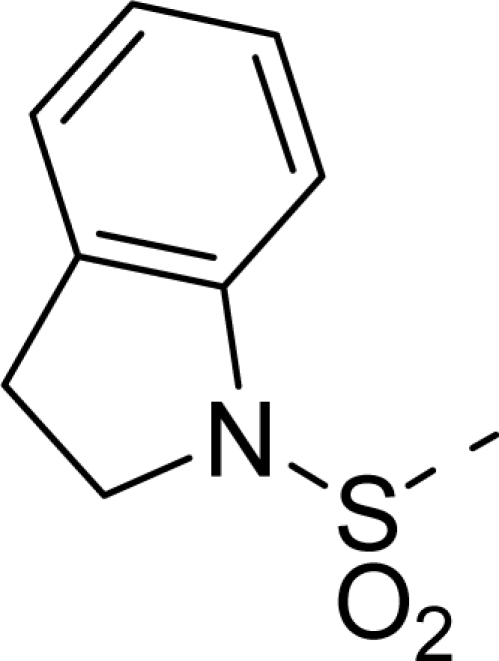	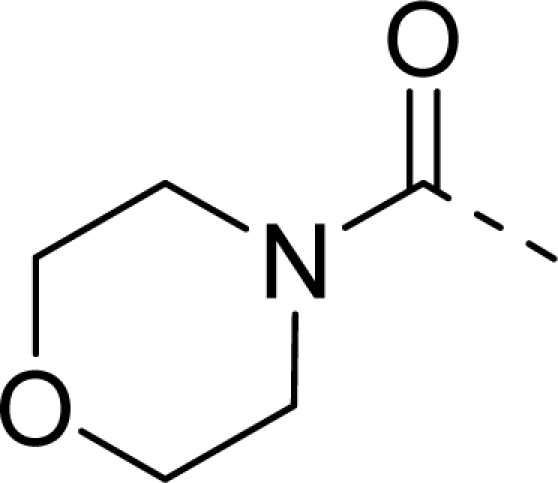
**31**	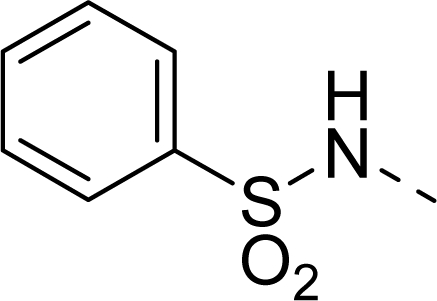	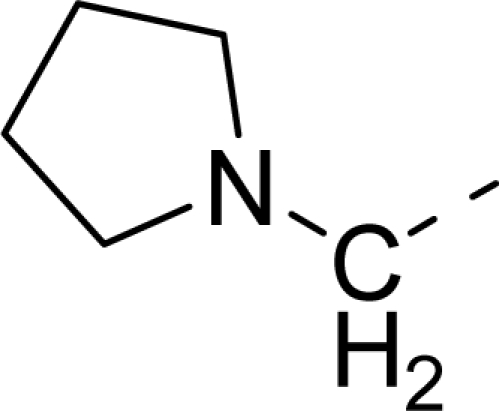
**32**	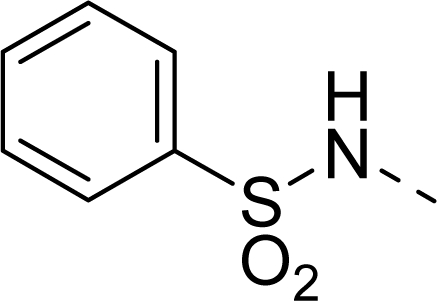	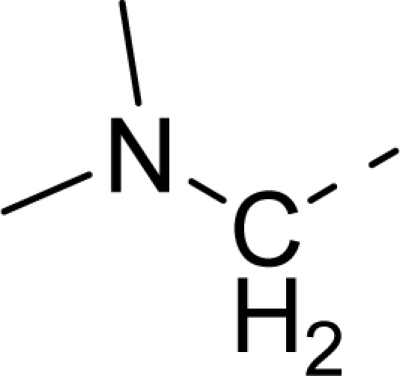
**33**	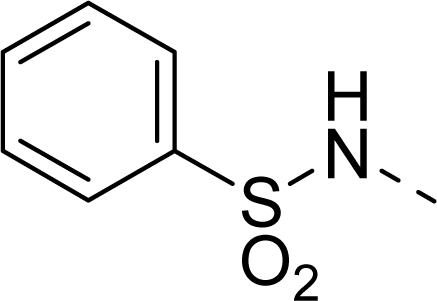	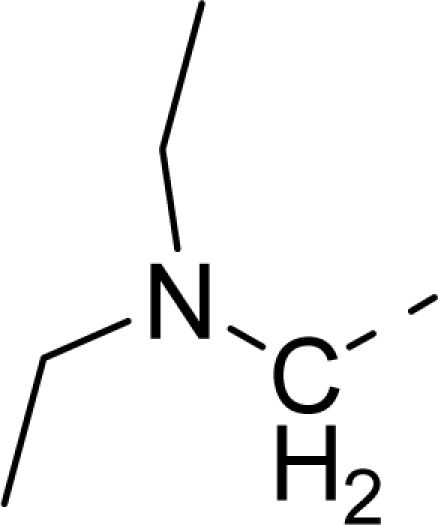
**34**	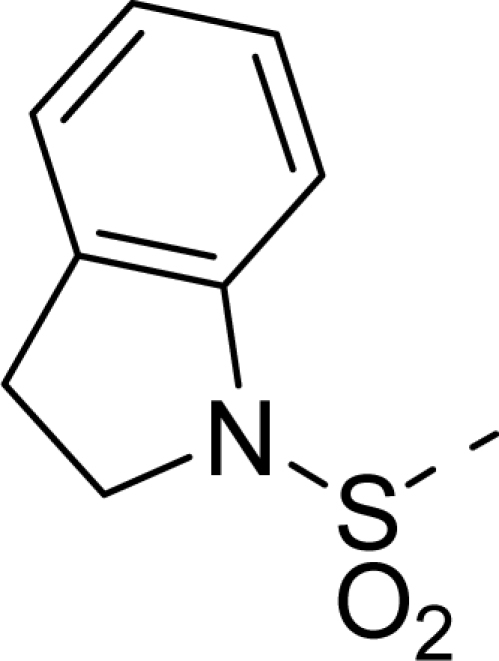	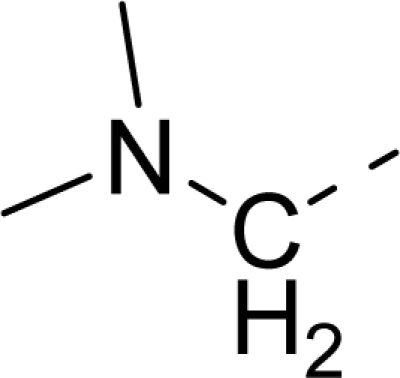
**35**	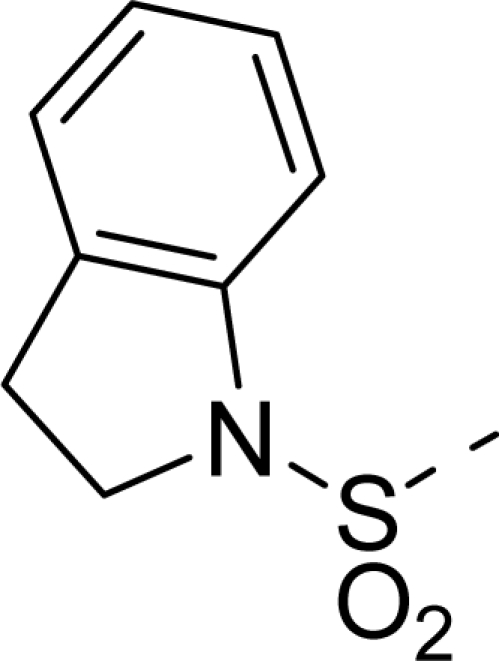	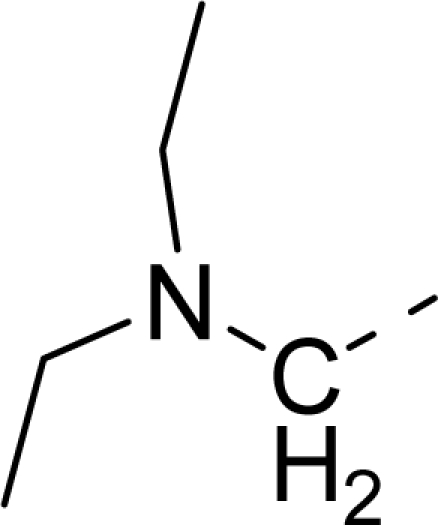

**Table 2.** The actual pIC_50_s, predicted pIC_50_s (Pred.) and their residuals (Res.) of the training and test set molecules.

**Compd. No.**	**pIC_50_**	**CoMFA**		**CoMSIA**	

	**Actual**	**Pred.**	**Res.**	**Pred.**	**Res.**
**1**	5.750	5.775	−0.025	5.703	0.047
2*	5.703	5.698	0.005	5.819	−0.116
**3**	6.682	6.573	0.109	6.512	0.170
4*	6.750	6.499	0.251	6.628	0.122
**5**	6.341	6.372	−0.031	6.510	−0.169
6*	6.441	6.536	−0.095	6.565	−0.124
**7**	6.678	6.602	0.076	6.729	−0.051
**8**	6.140	6.473	−0.333	6.175	−0.035
**9**	6.447	6.262	0.185	6.403	0.044
**10**	6.252	6.211	0.041	6.342	−0.090
11*	6.599	6.844	−0.245	6.471	0.128
**12**	6.143	6.221	−0.078	6.136	0.007
13*	7.244	7.333	−0.089	7.155	0.089
**14**	7.409	7.390	0.019	7.345	0.064
**15**	6.567	6.455	0.112	6.498	0.069
**16**	6.354	6.347	0.007	6.421	−0.067
**17**	7.215	7.209	0.006	7.152	0.063
**18**	7.509	7.580	−0.071	7.530	−0.021
19*	7.678	7.544	0.134	7.566	0.112
**20**	8.155	8.073	0.082	8.049	0.106
**21**	7.854	7.803	0.051	7.977	−0.123
**22**	7.538	7.623	−0.085	7.548	−0.010
**23**	7.469	7.715	−0.247	7.623	−0.155
**24**	7.921	7.623	0.298	7.751	0.170
25*	7.201	7.545	−0.344	7.449	−0.248
**26**	7.076	7.101	−0.025	6.995	0.081
**27**	6.662	6.630	0.032	6.806	−0.145
28*	6.551	6.697	−0.146	6.857	−0.306
**29**	6.932	7.030	−0.098	6.880	0.052
**30**	6.250	6.303	−0.053	6.350	−0.101
31*	5.664	5.765	−0.102	5.682	−0.019
**32**	5.660	5.621	0.039	5.651	0.009
**33**	5.611	5.584	0.027	5.619	−0.008
**34**	5.644	5.677	−0.033	5.550	0.094
35*	5.631	5.660	−0.029	5.466	0.165

* Test set molecules.

**Table 3.** Results of CoMFA and CoMSIA models.

**Statistics**	**CoMFA**	**CoMSIA**
*r*^2^_cv_^a^	0.726	0.566
*r*^2^^b^	0.972	0.984
ONC^c^	6	6
SEE^d^	0.144	0.110
F value^e^	105.300	181.398
*r*^2^_pred_^f^	0.937	0.948
Field contribution		
Steric	0.457	0.152
Electrostatic	0.543	0.169
Hydrophobic	-	0.243
H-bond Donor	-	0.183
H-bond Acceptor	-	0.252

a cross-validated correlation coefficient;

b non-cross-validated coefficient;

c optimal number of components;

d standard error of estimate;

e value F-test value;

f predictive correlation coefficient.

**Table 4.** Structure, predicted pIC_50_ values, and surflex-dock total score of the newly designed molecules.

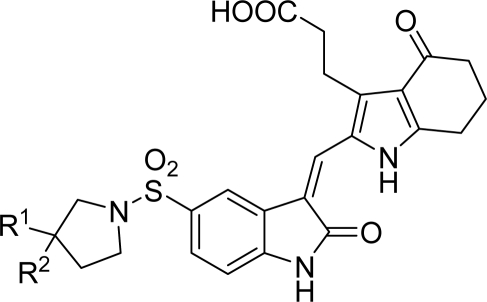					
**Compound NO.**	**Substituent**		**Predicted pIC_50_**		**Total score**

	**R^1^**	**R^2^**	**CoMFA**	**CoMSIA**	
**20**	H	H	8.073	8.049	8.49
**D1**	H	C(CH_3_)_3_	7.822	8.095	9.09
**D2**	H	CH_2_C(CH_3_)_3_	7.823	8.133	10.28
**D3**	H	CH_2_CH_2_C(CH_3_)_3_	8.044	8.302	9.82
**D4**	CH_3_	CH_3_	8.047	8.294	8.91
**D5**	H	CH_3_	8.091	8.203	7.60
**D6**	CH_3_	CH_2_CH_3_	7.710	8.160	8.88
**D7**	CH_2_CH_3_	CH_2_CH_3_	7.661	8.156	9.23
**D8**	CH_2_CH_2_CH_3_	CH_2_CH_2_CH_3_	7.647	8.145	10.46
**D9**	CH_3_	cyclohexane	7.633	8.154	10.32
